# Multilocus Analysis Resolves the European Finch Epidemic Strain of *Trichomonas gallinae* and Suggests Introgression from Divergent Trichomonads

**DOI:** 10.1093/gbe/evz164

**Published:** 2019-07-30

**Authors:** Abdulwahed Fahad Alrefaei, Ross Low, Neil Hall, Rodrigo Jardim, Alberto Dávila, Rick Gerhold, Shinto John, Sascha Steinbiss, Andrew A Cunningham, Becki Lawson, Diana Bell, Kevin Tyler

**Affiliations:** 1 School of Biological Sciences, University of East Anglia, Norwich, Norfolk, United Kingdom; 2 Department of Zoology, King Saud University, College of Science, Riyadh, Saudi Arabia; 3 Earlham Institute, Norwich Research Park Innovation Centre, Colney Ln, Norwich, United Kingdom; 4 Oswaldo Cruz Institute, Oswaldo Cruz Foundation, Rio de Janeiro, Brazil; 5 University of Tennessee, Center for Wildlife Health, Knoxville, Tennessee; 6 Institute of Zoology, Zoological Society of London, Regent's Park, London, United Kingdom; 7 Wellcome Trust Sanger Institute, Parasite Genomics, Hinxton, Cambridge, United Kingdom; 8 University of East Anglia, School of Medicine, Biomedical Research Centre, Norwich, Norfolk, United Kingdom

**Keywords:** trichomonosis, trichomoniasis, genome, molecular epidemiology, emerging infectious disease, wild bird, MLST, hybrid

## Abstract

In Europe, *Trichomonas gallinae* recently emerged as a cause of epidemic disease in songbirds. A clonal strain of the parasite, first found in the United Kingdom, has become the predominant strain there and spread to continental Europe. Discriminating this epidemic strain of *T. gallinae* from other strains necessitated development of multilocus sequence typing (MLST). Development of the MLST was facilitated by the assembly and annotation of a 54.7 Mb draft genome of a cloned stabilate of the A1 European finch epidemic strain (isolated from Greenfinch, *Chloris chloris*, XT-1081/07 in 2007) containing 21,924 protein coding genes. This enabled construction of a robust 19 locus MLST based on existing typing loci for *Trichomonas vaginalis* and *T. gallinae*. Our MLST has the sensitivity to discriminate strains within existing genotypes confidently, and resolves the American finch A1 genotype from the European finch epidemic A1 genotype. Interestingly, one isolate we obtained from a captive black-naped fruit dove *Ptilinopsus melanospilus*, was not truly *T. gallinae* but a hybrid of *T. gallinae* with a distant trichomonad lineage. Phylogenetic analysis of the individual loci in this fruit dove provides evidence of gene flow between distant trichomonad lineages at 2 of the 19 loci examined and may provide precedence for the emergence of other hybrid trichomonad genomes including *T. vaginalis*.

## Introduction

A clonal strain of *Trichomonas gallinae* has recently emerged as a cause of epidemic disease in passerines in Europe: Whilst this strain also causes trichomonosis in columbids and birds of prey, it is a minority strain in healthy columbids (Lawson, Cunningham, et al. 2011; Chi et al. 2013). This epidemic was first reported in finches in the United Kingdom (Pennycott et al. 2005). Since when there has been unprecedented large-scale mortality of greenfinches, *Chloris chloris*, which has led to a 66% reduction in the British breeding population; from a peak of circa 4.3 million when the disease first emerged, to circa 1.5 million individuals in 2016 (Lawson et al. 2018). Lethal spillover to other British passerines has also been observed (Robinson et al. 2010; Lawson, Robinson, et al. 2011). Since some British raptors feed on passerine species (Cotgreave 1995), concern has been raised regarding the potential for an increase in raptor mortality due to trichomonosis as a result of the finch epidemic (Chi et al. 2013). Since 2008, finch trichomonosis has been reported in southern Fennoscandia and continental Europe (Peters et al. 2009; Forzan et al. 2010; Neimanis et al. 2010; Ganas et al. 2014; Gourlay et al. 2014), with chaffinch migration believed to be the primary vector of spread (Lawson, Robinson, et al. 2011).

Sequence data from the 5.8S ribosomal RNA (rRNA) and surrounding internal transcribed spacer regions 1 and 2 (ITS1, ITS2) have been increasingly used to detect *T. gallinae* infection (Gaspar da Silva et al. 2007) and to identify genetic heterogeneities in the parasite (Felleisen 1997; Gerhold et al. 2008; Anderson et al. 2009; Grabensteiner et al. 2010). Sequence analyses of the ITS1/5.8S/ITS2 region (hereafter called ITS region) have identified marked variation amongst isolates obtained from a wide geographical region and from different host taxa, with some 15 distinct ITS region sequences identified as discrete ITS ribotypes (Gerhold et al. 2008; Anderson et al. 2009; Sansano-Maestre et al. 2009; Grabensteiner et al. 2010).

ITS ribotypes of *T. gallinae* parasites obtained from 11 species of affected British passerines showed that they had 100% identity to each other and to ITS ribotype A isolates from the United States (Gerhold et al. 2008; Anderson et al. 2009), Brazil (Kleina et al. 2004), Spain (Sansano-Maestre et al. 2009), and Austria (Grabensteiner et al. 2010). Using the (hydrogenosomal) iron hydrogenase (Fe-hydrogenase) gene as a second genotyping marker (one which is particularly useful for a mitochondrial protists that lack widely used mitochondrial house-keeping protein encoding genes; Voncken et al. 2002), we detected finer-scale genetic variation between *T. gallinae* sequences (Lawson, Cunningham, et al. 2011). Although we found no variation amongst British passerine samples at this locus, when we compared columbid and bird of prey isolates collected from the United Kingdom and elsewhere, marked sequence diversity at the Fe-hydrogenase gene was observed which was not detected from the ITS region alone (Chi et al. 2013). Thus we proposed a simple alphanumeric genotype with the letter being drawn from the ITS ribotype and the number from the Fe-hydrogenase subtype and in which A1 was the genotype of the European finch epidemic strain (Chi et al. 2013).

Recently the A1 genotype has also been reported as an emerging cause of trichomonosis outbreaks in wild finches in the Canadian Maritimes (McBurney et al. 2015) and as an infection of wild columbids in the United States (Girard et al. 2014). It is possible, therefore, that there are multiple strains of *T. gallinae* within the A1 genotype worldwide. In order to determine the transmission pathways of this parasite between bird species and the spread of the recently emerged strains causing finch trichomonosis outbreaks in Europe and Canada, tools to further discriminate between parasites strains are required.

Multilocus sequence typing (MLST) is a nucleotide sequence based method that is used to characterize the genetic relationships between microbial species. It has been successfully applied to study populations of bacterial and eukaryotic organisms (Maiden et al. 1998; Hanage et al. 2005; Tibayrenc 2009). Selected loci are normally single copy housekeeping genes so that the variation within these genes is nearly neutral but less prone to homoplasy than using an alternative approach such as multilocus microsatellite typing, and thus they are better able to serve as robust markers of ancient and modern ancestry.

Since the draft genome of the closely related *Trichomonas vaginalis* was completed, two overlapping sets of MLST loci have been proposed (Conrad et al. 2011; Cornelius et al. 2012). We have sought to extend these analyses by applying them to *T. gallinae* and have therefore produced a draft annotated genome of the European finch epidemic strain of *T. gallinae* and used it initially to identify the loci involved. Primers were then produced to enable a parallel 19 locus MLST analysis from nine isolates of *T. gallinae* from captive and wild birds for comparison with a *T. vaginalis* reference strain.

## Materials and Methods

### Isolation and Cloning of the Genome Clonal Stabilate GF1c

For the genome stabilate, *T. gallinae* was isolated from a greenfinch found dead with esophageal thickening, consistent with trichomonosis, in Norfolk in October 2007 (isolate XT1081-07) (table 1). A sample was taken from the infected bird during postmortem examination at the time of initial presentation and inoculated into Trichomonas Medium No. 2 (Oxoid, United Kingdom), incubated at 30 °C and screened for motile trichomonads at days 1, 2, and 5 (Robinson et al. 2010). The initial stabilate for the isolate was obtained and cryopreserved in liquid nitrogen with 5% dimethyl sulfoxide (DMSO) at −196 °C until further processing. This stabilate was subsequently revived and cultured axenically in pH 7.2 Trypticase-yeast extract-maltose (TYM) supplemented with 10% heat inactivated horse serum at 37 °C (Diamond 1954), parasites were subcultured three times prior to cloning. Cultures were diluted to 50 parasites per milliliter, so that one parasite could be found on average in every 2 (10 μl) wells of Terasaki plates (Greiner Bio-One, Kremsmünster, Austria). The parasites were allowed to settle to the bottom of the wells, the plates were then scanned using an inverted microscope, the positions of wells containing only a single motile parasite were recorded. Wells containing no parasites or more than one were noted and ignored for cloning purposes. The plates were incubated for 24 h at 37 °C in an atmosphere containing 5% carbon dioxide (CO_2_) at 80% relative humidity before wells where the parasites had replicated were used to inoculate larger cultures. Clones were subcultured three times in TYM and in one case, for the genome clonal stabilate GF1c, the cloning procedure repeated. Finally, clones were recultured and adjusted to a final concentration of 5 × 10^6^ living organisms per milliliter in TYM before cryopreservation. When in the logarithmic phase of growth, all cloned trichomonads exhibited normal morphology and more than 95% motility. GF1c was cryopreserved in liquid nitrogen with 5% DMSO at −196 °C. A clonal stabilate of the captive black-naped fruit dove isolate (BND1c) was subsequently produced by the same method as described for GF1c.

**Table 1:** List of Case Isolate ID, Bird Species, Year Found, Location, Evidence of Upper Alimentary Tract Lesions Consistent With Trichomonosis, Genotype

Case No.	Host Species	Year Found	Isolate Origin	Oropharyngeal Lesions	Genotype *
XT-1081/07 (GF1c)	Greenfinch *Chloris chloris*	2007	Norfolk, England Wild	Yes	A1 Europe
HF1	House finch *Haemorhous mexicanus*	2006	Kentucky, United States Wild	Yes	A1 United States
R11	Feral pigeon *Columba livia*	2004	Georgia, United States Wild	No	A2
R-1604/13 (BND1c)	Black-naped Fruit Dove *Ptilinopsus melanospilus*	2013	UK Zoological Collection Captive	No	M1
5 UEA	Feral pigeon *Columba livia*	2012	Norfolk, England Wild	No	C2
Fh49001	Woodpigeon *Columba palumbus*	2014	Norfolk, England Wild	Yes	C4
Norfolk31/15	Feral pigeon *Columba livia*	2015	Norfolk, England Wild	No	C8
Norfolk32/15	Feral pigeon *Columba livia*	2015	Norfolk, England Wild	No	C9
R-138/14	Socorro dove *Zenaida graysoni*	2015	UK Zoological Collection Captive	No	C10

Note.—Location refers to United Kingdom counties and United States state where the bird was found. *Genotyping scheme according to Chi et al. 2013.

### Preparing DNA Extraction for Whole Genome (Illumina) Sequencing

Extraction of *T. gallinae* genomic DNA (gDNA) from parasite cultures was performed using DNAzol (Invitrogen, United Kingdom) essentially as described in the manufacturer’s instructions. A TruSeq PCR-Free kit was used with the gDNA to generate an Illumina shotgun library (insert size 200–300 bp). Paired-end sequencing (2 × 150 bp) was performed at the Centre for Genomic Research, University of Liverpool, on an Illumina HighSeq 2500 platform. The draft genome sequence was assembled de novo using Velvet (Zerbino and Birney 2008). The theoretical size of the genome was estimated from k-mer frequencies calculated in kAT (Mapleson et al. 2017) and shown in supplementary figure S1*B*, Supplementary Material online. Genome size was calculated as the total number of k-mers (area under the curve) divided by the coverage (mean coverage/curve peak), using the R statistical package. The peak was also compared with a Poisson distribution (supplementary fig. S1*C*, Supplementary Material online). Genome assembly quality was assessed using a k-mer spectra plot generated in kAT by comparing the k-mer content between the final assembly and the trimmed paired end reads using a k-mer size of 27 (supplementary fig. S1*A*, Supplementary Material online).

### A New Draft Trichomonad Genome

A total of 32,936,526 paired-end reads were generated from sequencing, which were adapted and quality trimmed using cutadapt (version 1.2.1). The draft genome sequence was assembled de novo using a version of SPAdes. We used a homology-based approach with the aim of annotating the draft genome of *T. gallinae* using the OrthoMCL software (version 1.4) (Li et al. 2003) via shared gene cluster membership after including genomic data from closely related organisms. Initially we downloaded 541 coding sequences (CDS) of *T. vaginalis* genes from RefSeq to prepare a model for use with the GlimmerHMM software (Majoros et al. 2004). Using this model, we predicted 22,348 open reading frames (ORFs) in the contigs of the *T. gallinae* genomic sequence. Finally, we performed an OrthoMCL analysis of these ORFs against the *T. vaginalis* proteome downloaded from RefSeq (Carlton et al. 2007). Annotations for 16,651 ORFs (74.51%) were transferred from *T. vaginalis*, with 10,444 genes (46.73%) being annotated as hypothetical genes. It should be noted, that while the *T. vaginalis* draft genome contains 59,681 predicted genes, only about 26,000 are supported by experimental evidence (which is corroborated by the number of genes found in our study); the same study suggesting that the *T. vaginalis* genome contains large gene families and many repetitions (Carlton et al. 2007). This situation would make overcompression of the draft genome, by grouping repeated reads in the same contig, more likely, hence decreasing the apparent size of the genome.

To assess the completeness of our draft genome and annotation BUSCO analysis was used to assess the number of Benchmarking Universal Single-Copy Orthologs. The results shown in table 2 show that the draft genome and annotation are high quality and comparable. Overall the *T. gallinae* annotation has fewer fragmented BUSCOs than the *T. vaginalis* annotation despite the large difference in predicted proteome sizes. The difference between the genome and annotation scores stems from the protein coding prediction method employed by BUSCO, which is not trained for specific genomes. The fact that not all of the BUSCOs are present may be attributed to the BUSCOs that were searched for being generic eukaryote BUSCOs rather than specific for Trichomonads. Collinearity between *T. vaginalis* and *T. gallinae* was determined for contigs and predicted CDS’s using the MUMmer programs NUCmer and PROmer, respectively. Results were visualized and plotted using mummerplot (Delcher et al. 2003).

**Table 2:** BUSCO Reports for *T. gallinae* and *T. vaginalis* Giving a Measure of the Completeness of the Genome and Showing that the *T. gallinae* Genome and Annotation is Comparable to the *T. vaginalis* Genome

BUSCO Content	*T. gallinae* Genome	*T. gallinae* Proteome	*T. vaginalis* Genome	*T. vaginalis* Proteome
Complete	186	207	186	209
Single copy	163	158	154	160
Duplicated	23	49	32	49
Fragmented	13	9	15	16
Missing	104	87	102	78
Completeness (%)	65.67	71.28	66.33	74.25

### Parasite Strains and DNA Extraction for MLST

Eight additional reference strains (table 1) of *T. gallinae* from captive and wild birds from the United Kingdom and United States, collected between 2007 and 2015, were analyzed using MLST in addition to the GF1c isolate. Most of these strains have been widely used in previous studies (Gerhold et al. 2008; Lawson, Cunningham, et al. 2011; Chi et al. 2013). Cryopreserved isolates were revived and cultured in modified TYM medium at 37 °C and gDNA was extracted as per GF1c (above).

### Choice of Loci for MLST Typing

Using the whole genome sequence of GF1c, genes were verified as single copy by performing an all-against-all, basic local alignment search tool (BLAST) comparison of nucleotide sequences. Based on their diversity and their ability to be readily amplified and sequenced on both DNA strands, 19 housekeeping loci were identified for the final MLST scheme. Each of the 19 loci was on a different contig of the sequenced genome. Primers were designed for each of the 19 loci using Primer-BLAST at the GenBank website (http://www.ncbi.nlm.nih.gov/tools/primer-blast/; last Accessed August 1, 2019). All primers were designed from common sequence for the *T. gallinae* genes and *T. vaginalis* orthologs, using our new draft genome and the published draft genome sequence of *T. vaginalis* (version 1.2, http://trichdb.org; last Accessed August 1, 2019). 

### PCR Amplification for MLST

Primers for the sequencing of MLST loci were designed to amplify gene fragments of 655–966 base pairs in length. Details of primers and reaction conditions are provided in supplementary table S1, Supplementary Material online. All genes were amplified as follows: 30 μl PCR reactions containing 20 μl of HotStarTaq Master Mix Kit (Qiagen, Inc., United Kingdom), 2 μl of distilled water, 3 μl of forward primer, 3 μl of reverse primer, and 2 μl of template of DNA. We used the same thermocycler program parameters for all loci as follows: Initial denaturation of template DNA at 95 °C for 3 min; 40 cycles of 95 °C for 45 s, 55–57 °C for 1 min, 72 °C for 2 min with a 5-s extension after each cycle, followed by the final extension at 72 °C for 7 min. Samples were visualized on 1% agarose gels to verify amplification. Bidirectional Sanger sequencing was conducted by Source BioScience (Nottingham, United Kingdom). Sequence alignments were carried out using MEGA software version 6.06.

### Sequence Analysis of MLST and Phylogenetic Trees

The nucleotide sequence of each of the selected housekeeping genes from *T. gallinae* was aligned with its respective gene sequence from the nine *T. gallinae* isolates using maximum likelihood (ML) trees generated using Molecular Evolutionary Genetics Analysis (MEGA) software version 6.06 (Tamura et al. 2011), both for individual genes and for the concatenated gene sequences. Felsenstein’s bootstrap test was used to evaluate the support for tree topologies and clustering of taxa (2,000 times) (Felsenstein 1985).

For the phylogenetic tree based on the housekeeping genes, ML tree evolutionary distances were computed using the Tamura-Nei method in units of the number of base substitutions per site (Tamura and Nei 1993). Bootstrap values of the ML tree with the highest log likelihood were obtained. For all trees, all positions containing gaps and missing data were eliminated. Constrained and unconstrained ML trees were used to test topological significance (supplementary fig. S*3* and table S3, Supplementary Material online). Trees were built using GTR model and Gamma correction in iqtree (Nguyen et al. 2015). Evolutionary analyses were conducted using MEGA 6.06 (Tamura et al. 2011) and network analyses were performed using Splitstree (Huson and Bryant 2006) to construct a Neighbor-Net analysis from catenated MLST fragments after which a final consensus network, obtained from the combination of the individual ML tree for each gene, was constructed. These networks were also used to test for evidence of recombination using the Pairwise Homoplasy Index, which is included in the Splitstree package.

## Results

### Isolates Used

For this study, we investigated the potential for increased resolution offered by MLST. Nine isolates of *T. gallinae* of known genotype were selected for the study; seven of the isolates were obtained in the United Kingdom (five wild birds and two captive), and two from wilds birds in the United States (table 1). Three isolates were ribotype A, one subtype A2, two subtype A1; one A1 was isolated from a UK greenfinch and the other from a US house finch (*Haemorhous mexicanus*). Five of the isolates, from captive and wild columbids in the United Kingdom, were ribotype C. One isolate was a highly divergent ribotype M, which was isolated in the United Kingdom from a captive-bred black-naped fruit dove.

### Genome Sequence Data

MLST genes were selected based on the previously validated MLST of *T. vaginalis* proposed by Conrad et al. (2011). Initially, we attempted amplification and sequencing using the published *T. vaginalis* primers, however, few of the loci amplified using template DNA from *T. gallinae* isolates (data not shown). To engineer our own primers for *T. gallinae* orthologs, we produced a draft genome sequence of *T. gallinae* which we present here, the draft genome sequence and annotation of a clone from an isolate of the European finch epidemic strain of *T. gallinae* (A1) GF1c (GenBank accession number MRSU00000000).

The final assembly of the *T. gallinae* draft genome sequence was determined to consist of 11,704 contigs and an N50 contig size of 20,741. The assembly size was 54,799,485 bp with a coverage of 90× and a G + C content of 33.77%. The BUSCO analysis suggested a completeness comparable to the published *T. vaginalis* draft genome (table 2). The annotation results revealed 22,251 predicted genes, and 21,924 protein-coding sequences, including three pseudogenes and four rRNAs. There were also 319 transfer RNAs (tRNAs) including predicted tRNAs for all amino acids (table 3).

**Table 3:** Summary for the *Trichomonas gallinae* GF1c genome assembly (GenBank Accession Number MRSU00000000)

Feature	Value
Genome	
Size of assembly (bp)	54,799,485
Gene G + C content (%)	38.77
No. of scaffolds	11,704
N50 scaffold size (bp)	20,741
Protein-coding genes	
No. of predicted genes	21,924
Mean gene length (bp)	1,392
Gene G + C content (%)	38.25
Gene density (genes/Mb)	400.08
Nonprotein-coding genes	
No. of nonprotein-coding genes	327
Predicted tRNA genes	319
Ribosomal RNA (rRNA)	4
Predicted snRNAs	4

**Fig. 1.**
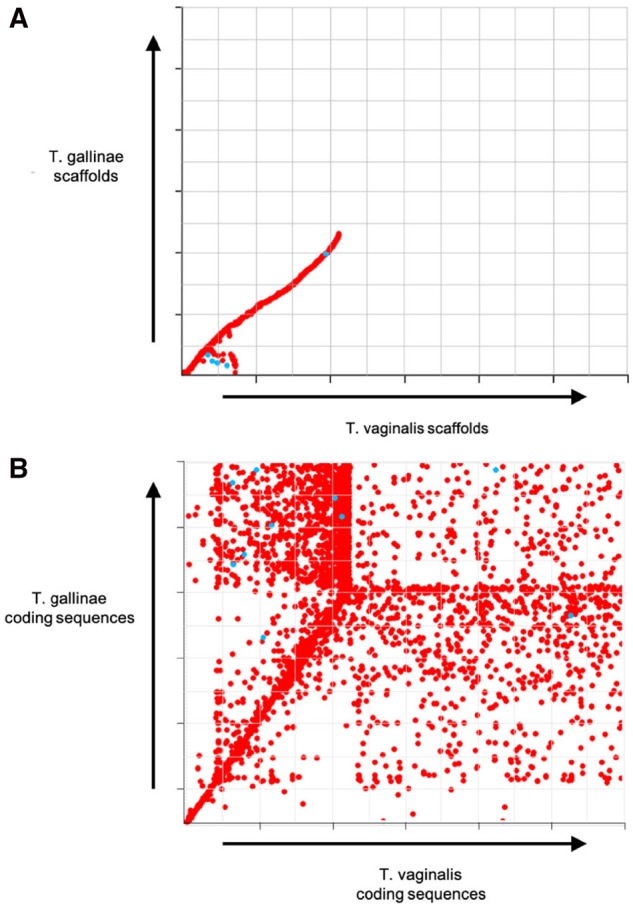
—MUMmer plot showing collinearity between *Trichomonas gallinae* and *Trichomonas vaginalis.* Red points denote collinearity matches >75% sequence identity and blue matches are inverted for both the contigs (*A*) and the coding sequences (*B*) for *T. gallinae* and *T. vaginalis*.

To test for collinearity between the *T. vaginalis* and *T. gallinae* assemblies, the contigs were plotted against each other in MUMmer (fig. 1*A*). This analysis revealed collinearity between 667 unique *T. vaginalis* contigs and 1,487 unique *T. gallinae* contigs representing 1% and 12% of the total number of contigs, respectively. Given this low proportion of collinearity between contigs, the predicted coding sequences of *T. vaginalis* and *T. gallinae* were tested as well (fig. 1*B*). This showed 1,585 coding sequences in *T. vaginalis* are colinear with 13,551 coding sequences in *T. gallinae* representing 2.7% and 61% of the total number of coding sequences, respectively.

### MLST Improves Strain Resolution and Discriminates the American A1 Strains from the European Finch A1 Epidemic Strain

The whole genome data were successfully mined for the *T. gallinae* MLST orthologous genes. Primers were then designed to amplify a long internal fragment of each gene. All 19 housekeeping gene fragments were successfully amplified using PCR and were sequenced for each of the *T. gallinae* isolates (table 5; supplementary table S1, Supplementary Material online) affording comparison of 14,770 bp, and yielding 2,043 discriminating characters of 19 concatenated sequences for all 9 isolates of *T. gallinae* and the single *T. vaginalis* isolate.

**Table 4:** Multilocus Sequence Typing (MLST) Loci Employed

*T. gallinae*Gene ID	*T. vaginalis*Gene ID	Locus (supplementary fig. S2, Supplementary Material online) label	Gene Name	Length	SNPs	Nucleotides per SNP
TGA-000149300	TVAG-087140	A	*Arp2/3*, putative	808	4	202
TGA-001385000	TVAG-400860	B	Clan MA, family M8, leishmanolysin-like metallopeptidase (*GP63a*)	682	16	43
TGA-00112400	TVAG-005070	C	Mismatch repair MutL homolog (*MIh1A*)	708	59	12
TGA-000731500	TVAG-302400	D	Mismatch repair MutL homolog (*MIh1A*)	966	32	30
TGA-000149500	TVAG-021420	E	Coronin (*CRN*)	904	18	50
TGA-000080800	TVAG-364940	F	Antigenic protein P1, putative (*VSA*)	741	71	10
TGA-002154000	TVAG-216430	G	Clan MA, family M8, leishmanolysin-like metallopeptidase (G*P63b*)	771	53	15
TGA-001611300	TVAG-303420	H	Vesicular mannose-binding lectin, putative (*LLF4*)	725	36	20
TGA-000024800	TVAG-291830	I	Vesicular mannose-binding lectin, putative, PS(*LLF1*)	736	32	23
TGA-000367600	TVAG-228710	J	Clan CA, family C1, cathepsin L-like cysteine peptidase	817	53	15
TGA-000478600	TVAG-485880	K	Clan CA, family C1, cathepsin L-like cysteine peptidase	721	80	9
TGA-001175900	TVAG-171780	L	HIV-1 rev binding protein, putative	759	53	14
TGA-001325800	TVAG-086190	M	Vesicular mannose-binding lectin, putative	910	40	28
TGA-002155200	TVAG-291970	N	Multidrug resistance pump, putative	743	50	15
TGA-000818700	TVAG-459080	O	Aspartic peptidase	900	29	31
TGA-000730800	TVAG-414100	P	Tropomyosin isoforms1/2, putative	655	33	20
TGA-000739900	TVAG-192620	Q	Actin depolymerizing factor, putative	657	31	21
TGA-001849400	TVAG-094560	R	Clan CE, family C48, cysteine peptidase	867	52	17
TGA-001506800	TVAG-309150	S	Conserved hypothetical protein (with PF03388 Domain)	700	31	23

The size of the fragments analyzed for the selected housekeeping genes ranged between 655 bp (TGA-000730800) and 966 bp (TGA-000731500) (cf. table 4). For each *T. gallinae* isolate, the sequence obtained for each of the 19 loci was compared with that of every other isolate, and single nucleotide polymorphisms (SNPs) were numbered consecutively. In total 773 *T. gallinae* SNPs were detected across these loci from 14,770 nucleotides analyzed, corresponding to an average of 1 SNP every 19 bp. The number of SNPs varied for each gene, ranging from 1 SNP every 9 bp for TGA-000478600 to 1 SNP every 202 bp for TGA-000149300 (table 4).

**Table 5:** List of GenBank Accession Numbers for Each Locus of the MLST

GenotypeGene ID	A1	A1_US	A2	M1	C2	C4	C8	C9	C10
TGA-000149300	MK550746	MK550747	MK550748	MK550749	MK550750	MK550751	MK550752	MK550753	MK550754
TGA-001385000	MK550836	MK550837	MK550838	MK550839	MK550840	MK550841	MK550842	MK550843	MK550844
TGA-00112400	MK550737	MK550738	MK550739	MK550740	MK550741	MK550742	MK550743	MK550744	MK550745
TGA-000731500	MK550791	MK550792	MK550793	MK550794	MK550795	MK550796	MK550797	MK550798	MK550799
TGA-000149500	MK550755	MK550756	MK550757	MK550758	MK550759	MK550760	MK550761	MK550762	MK550763
TGA-000080800	MK550728	MK550729	MK550730	MK550731	MK550732	MK550733	MK550734	MK550735	MK550736
TGA-002154000	MK550872	MK550873	MK550874	MK550875	MK550876	MK550877	MK550878	MK550879	MK550880
TGA-001611300	MK550854	MK550855	MK550856	MK550857	MK550858	MK550859	MK550860	MK550861	MK550862
TGA-000024800	MK550719	MK550720	MK550721	MK550722	MK550723	MK550724	MK550725	MK550726	MK550727
TGA-000367600	MK550764	MK550765	MK550766	MK550767	MK550768	MK550769	MK550770	MK550771	MK550772
TGA-000478600	MK550773	MK550774	MK550775	MK550776	MK550777	MK550778	MK550779	MK550780	MK550781
TGA-001175900	MK550818	MK550819	MK550820	MK550821	MK550822	MK550823	MK550824	MK550825	MK550826
TGA-001325800	MK550827	MK550828	MK550829	MK550830	MK550831	MK550832	MK550833	MK550834	MK550835
TGA-002155200	MK550881	MK550882	MK550883	MK550884	MK550885	MK550886	MK550887	MK550888	MK550889
TGA-000818700	MK550809	MK550810	MK550811	MK550812	MK550813	MK550814	MK550815	MK550816	MK550817
TGA-000730800	MK550782	MK550783	MK550784	MK550785	MK550786	MK550787	MK550788	MK550789	MK550790
TGA-000739900	MK550800	MK550801	MK550802	MK550803	MK550804	MK550805	MK550806	MK550807	MK550808
TGA-001849400	MK550863	MK550864	MK550865	MK550866	MK550867	MK550868	MK550869	MK550870	MK550871
TGA-001506800	MK550845	MK550846	MK550847	MK550848	MK550849	MK550850	MK550851	MK550852	MK550853

**Fig. 2.**
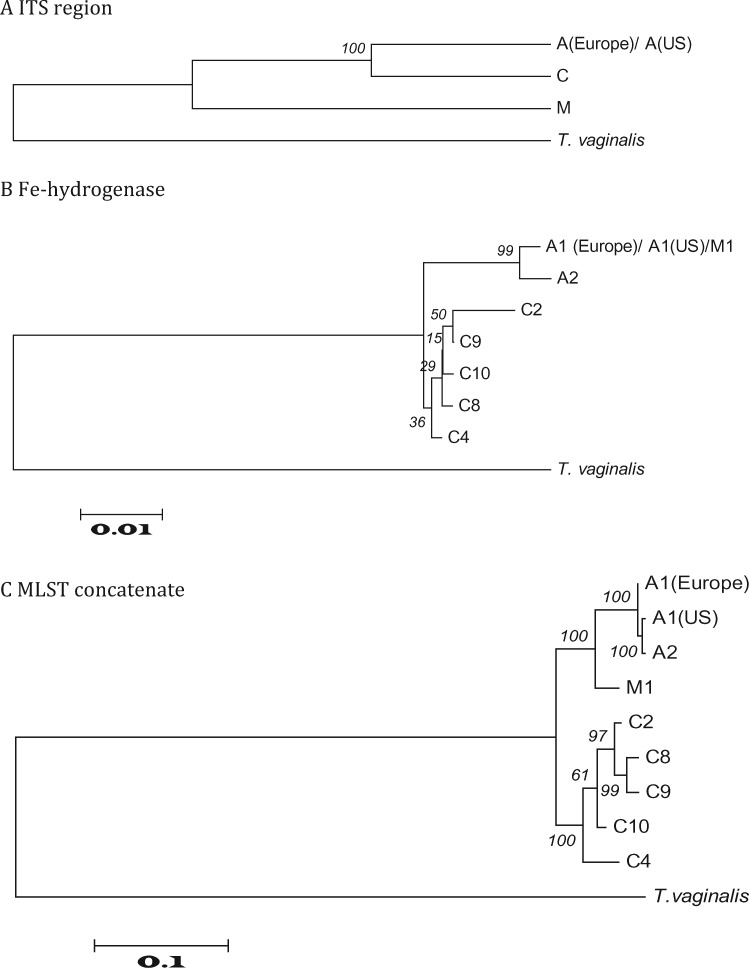
—A concatenated phylogeny of *Trichomonas gallinae* based on 19 housekeeping genes of MLST created by using maximum likelihood provides resolution for all strains tested. *Trichomonas vaginalis* is included as an outgroup. Numbers on branches indicate bootstrap values based on 2,000 replicates. (*A*) ITS ribotypes alone resolve only 3 groups of avian trichomonads A, C, and M. (*B*) Fe-hydrogenase is able to resolve 8 genotypes from the 10 avian trichomonads strains tested. (*C*) MLST based on 19 loci (listed in table 3) provides confident resolution of all the avian trichomonad strains tested. A1 (epidemic) encompasses isolates of the European finch epidemic A1 strain.

We used our panel of nine isolates and *T. vaginalis* as the outgroup to demonstrate that strains of *T. gallinae* are only partially resolved into three groups by ribotyping (fig. 2*A*) or seven groups using genotyping with Fe-hydrogenase (fig. 2*B*). However, our MLST sequences yield a tree with enough resolution to discriminate all nine of the isolates while providing for reduced variance in distance estimates. Our ML tree (fig. 2*C*) was based on 14,770 bp of concatenated sequence comprising all 19 loci. The tree was rooted with *T. vaginalis* as the outgroup. On this tree two groups of *T. gallinae* are evident*.* All of the ribotype C isolates cluster together, although it is notable the C4 subtype is significantly divergent from the other ribotype C isolates. The ribotype A isolates cluster with the ribotype M isolate. Notably the UK and US A1 subtype isolates are resolved by the MLST and, importantly, the US A1 genotype appears to be more closely related to the US A2 genotype in the panel than to the European finch epidemic strain A1 genotype, supporting different origins for these strains.

### Investigation of the M Isolate

The M isolate was cloned to ensure that the results obtained were not the result of a mixed infection. Both isolate and clone were found to be identical to the European finch epidemic strain at several loci, including the Fe-hydrogenase subtyping locus suggesting recent divergence from a common ancestor. The isolate is resolved in the concatenated tree because of the considerable divergence of a few loci. This incongruence is indicative of a hybrid origin and caused us to consider each of the MLST loci for evidence of introgressed genes.

Phylogenetic trees of individual genes were constructed using the ML and neighbor joining (NJ) methods. The tree topologies generated by these two methods were found to be entirely congruent. Individual phylogenetic trees for each of the 19 genes are presented in supplementary figure S2*A*–*S*, Supplementary Material online. Most of the coding genes (16/19) produced trees which were congruent with each other and with the Fe-hydrogenase subtyping gene, however, four loci (A, D, I, and the ITS region) produced incongruent trees. These four loci fell into two congruent groups: First, the D and I alleles were congruent with each other, showing greater similarity to alleles from ribotype C isolates than to other ribotype A isolates. Second, the trees produced from the ITS region and from A were congruent with each other but the M isolate gene sequences diverged substantially from those of the other isolates, suggesting that they had a non-*T. gallinae* origin.

**Fig. 3.**
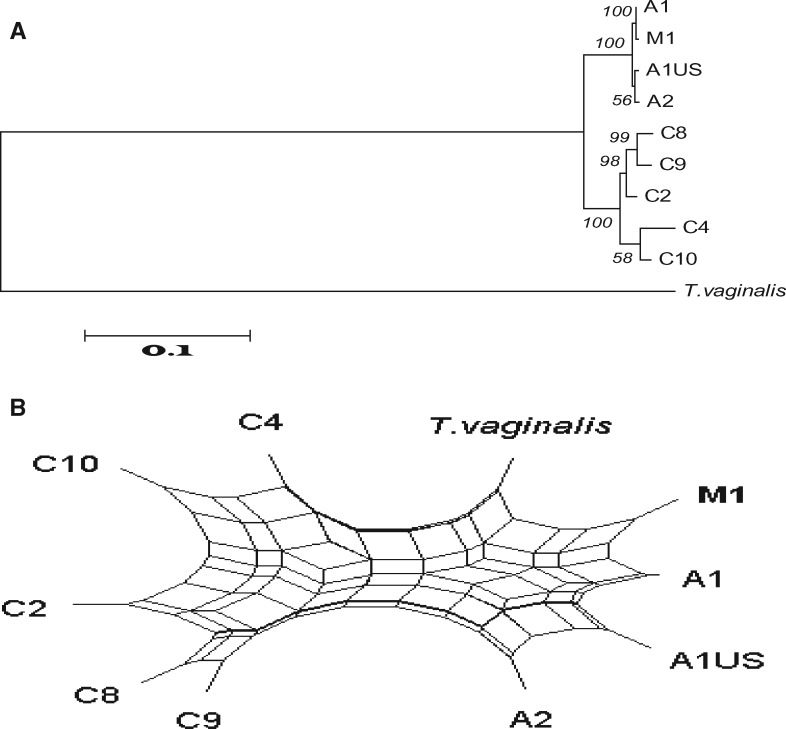
—Most markers group the M1 isolate with the A1 (European finch epidemic A1 strain). Phylogenetic trees and split network tree based on concatenated sequence analysis of *Trichomonas gallinae* strains, with 16 loci of MLST (all except loci A, D, and I) and the Fe-hydrogenase gene.

**Fig. 4.**
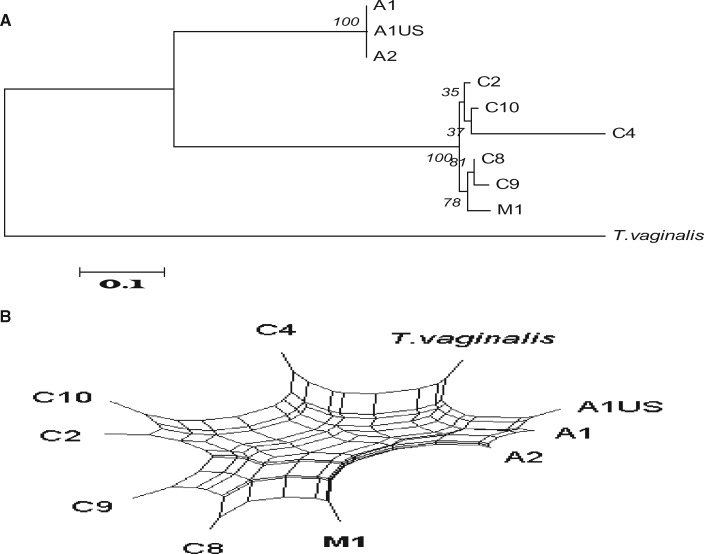
—Introgression of *Trichomonas gallinae* genes. The phylogenetic (*A*) and split network (*B*) trees based on concatenated sequence of Loci D and I are incongruous with other loci grouping the M1 isolate with C8 and C9 strains.

**Fig. 5.**
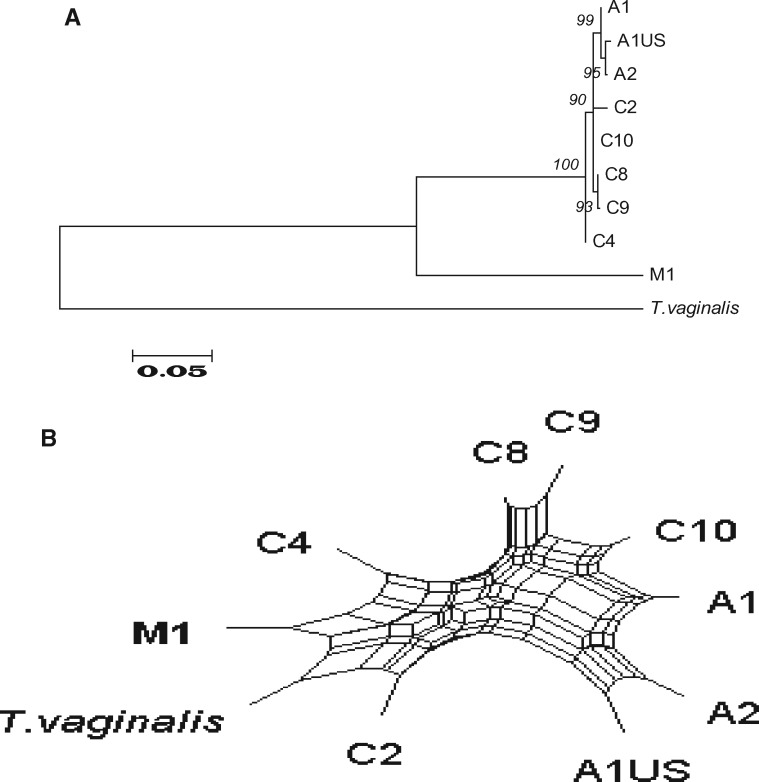
—Introgression of non-*Trichomonas gallinae* Trichomonad genes. The phylogenetic (*A*) and split network (*B*) trees based on concatenated sequence of Locus A and the ITS region are incongruous with other loci placing the M1 isolate outside of the *T. gallinae* group and thus suggesting a divergent trichomonad as the origin or these two loci.

We used trees and networks to further investigate the incongruent phylogenetic signals. Across all of our *T. gallinae* isolates, we compared 1) concatenated MLST, ITS region, and Fe-hydrogenase sequences (fig. 2); 2) with those from the congruent group of 16 loci (fig. 3), from D and I (fig. 4) and from A and the ITS region (fig. 5). We plotted the phylogenetic relationships using Neighbor-Net split network with the EqualAngle algorithm, which takes recombination (as evidenced for 6 of the MLST loci in supplementary table S2, Supplementary Material online) into account in formulating the network depictions. As evidenced in figure 3, the M1 isolate possesses alleles that segregate it with ribotype A isolates. Indeed, at most loci, it is indistinguishable from the A1 European finch epidemic strain. In contrast, the ITS ribotype and *Arp2/3* gene locus are so divergent that they do not group the isolate with any of the other isolates examined. Notably though, at two loci the alleles group most closely with C ribotype isolates C8 and C9 on the Neighbor-Net, but show some association with isolates of ribotype A—suggestive of gene flow between these groups.

## Discussion

### Improved Genotyping and Distinct Origins for UK and US Lineages of A1 Subtype

The 19 locus test described in this study demonstrated excellent consistency and discriminatory ability for assessing phylogenetic relationships for the *T. gallinae* isolates examined. These findings suggest that this MLST will be a valuable alternative to established genotyping and subtyping targets for *T. gallinae*. However, the test’s stability should be further assessed against a larger collection of isolates from different subtype strains and geographical locations to validate their discriminatory power.

Finch trichomonosis in the United States has been previously reported (Anderson et al. 2010) and identification of genotype A1 has also been reported from the United States in band-tailed doves—*Patagioenas fasciata* (Girard et al. 2014). Here, we are able to differentiate the American A1 strain from a house finch, from the A1 strain causing the European finch epidemic. It is of interest that the American A1 strain actually appears to be more closely related to the American A2 strain, than the European A1 strain, suggesting some modest homoplasy may occur in Fe-hydrogenase alleles.

### Evidence for New Strain Emergence from Trans-Species, Introgressed Hybrids

Our phylogeny based on the ITS region (fig. 2*A*) shows that the isolate from a black-naped fruit dove possessed a distinct M ribotype which was highly divergent from the *T. gallinae* complex. This suggested that the black-naped fruit dove parasite is a novel trichomonad species. Surprisingly, when subtyped with the Fe-hydrogenase gene the result was incongruent and the sequences obtained were identical to the A1 genotype of the European finch epidemic strain.

An obvious possibility to reconcile the results was that DNA had been extracted from a mixed infection, hence the black-naped fruit dove isolate was cloned. Despite being derived from a single parasite, the initial sequencing results were reproduced, namely a novel and highly divergent M ribotype with an A1 genotype sequence for the Fe-hydrogenase locus.

Using all MLST markers together, a close relationship between the M ribotype and the European finch epidemic A1 genotype is clear. Considered individually, however, the markers used fell into three groups, each giving a discrete tree topology. Within each group for each single marker the tree topologies were congruent. The majority of loci (17 of 21) were identical or nearly identical to the sequence to the European finch epidemic A1 genotype and hence produced a tree which suggested that the majority of the genome could be considered to be A1. Two markers were clearly still *T. gallinae* alleles, but appeared to be more akin to isolates with C ribotypes, suggesting a recent cross between the European finch epidemic A1 strain with an as yet uncharacterized C type followed by introgression. Most intriguingly, two alleles appeared to be from a non-*T. gallinae* trichomonad: The ITS 5.8S ribosomal locus, as previously noted, and the locus encoding an actin related protein—two well conserved house-keeping genes. The implication of this result is that the parasite was not an entirely newly discovered diverged species, but rather a recent chimaera or hybrid between *T. gallinae* and a hitherto uncharacterized trichomonad species arising either by fusion of two distant lineages or by some manner of gene transfer. The black-naped fruit dove originates from Indonesia, Malaysia and the Philippines: It is possible that the loci from a divergent trichomonad species originated from the species’ native range. Indeed, it is noteworthy that other authors have identified evidence of a divergent trichomonad species in another frugivorous columbid from Australia, the Pied Imperial pigeon (*Ducula bicolor*) (Peters, 2013). The black-naped fruit dove from which the clonal isolate was derived was a captive bird from a UK zoological collection, whose parents were captive bred in a zoological collection in mainland Europe. It is therefore plausible that coinfection with multiple trichomonad parasite lineages, perhaps from different regions, occurred and led to hybrid formation.

*Trichomonas vaginalis* is morphologically indistinguishable from *T. gallinae* and it has been observed that some avian *Trichomonas* at least show high levels of sequence identity at some loci to *T. vaginalis* (Gerhold et al. 2008) prompting speculation as to the relationship of human and avian trichomonads (Maritz et al. 2014). It is therefore somewhat surprising to discover such little collinearity between the two draft genomes of these trichomonads. However, this does inform the results from the MLST analysis, not least by suggesting that the *T. gallinae* genome appears to be congruent with *T. vaginalis* over only a part of the total genome. We also show that there are small-scale repeats in the *T. vaginalis* draft genome that appear only once in the *T. gallinae* draft genome, coupled with the difference between the contig collinearity and the coding sequences this suggests that the majority of the genome where there is no collinearity is noncoding.

Our results indicate that the black-naped fruit dove isolate of the M strain shows evidence of introgression from two crosses one of which was likely to have been recent. On this basis, we speculate that occasional hybrid formation and introgression may be an important factor in allowing rapid colonization of new hosts as may have happened in the natural history of *T. vaginalis.* Future work utilizing high quality genomes and bespoke bioinformatic methods should help discriminate introgression from the possibility of lateral gene transfer, identify how and when and between what lineages genomic exchanges have occurred and elucidate the frequency of type of genetic exchange, particularly between more closely related lineages.

## Supplementary Material

Supplementary data are available at *Genome Biology and Evolution* online.

## Supplementary Material

evz164_Supplementary_Data

## Notes

**Data deposition:** This Whole Genome Shotgun project has been deposited at DDBJ/ENA/GenBank under the accession MRSU00000000. The version described in this paper is version MRSU01000000.
