# Supplementary material for: Multilocus Analysis Resolves the European Finch Epidemic Strain of Trichomonas gallinae and Suggests Introgression from Divergent Trichomonads
Source: Genome Biol Evol. 2019 Jul 30;11(8):2391–402. doi: 10.1093/gbe/evz164 (PMC6735722; doi:10.1093/gbe/evz164)
Supplement: evz164_Supplementary_Data [file evz164_supplementary_data.zip › Figure S2.docx]

Figure S2 (with outgroup)

| A    TGA-000149300 | B    TGA-001385000 |
| --- | --- |
| C    TGA-00112400 | D  TGA-000731500 |
| E    TGA-000149500 | F    TGA-000080800 |

| G    TGA-002154000 | H    TGA-001611300 |
| --- | --- |
| I    TGA-000024800 | J    TGA-000367600 |

| K    TGA-000478600 | L    TGA-001175900 |
| --- | --- |
| M    TGA-001325800 | N    TGA-002155200 |
| O    TGA-000818700 | P    TGA-000730800 |
| Q    TGA-000739900 | R    TGA-001849400 |
| S   TGA-001506800 |  |

No outgroup

| A    TGA-000149300 | B    TGA-001385000 |
| --- | --- |
| C    TGA-00112400 | D    TGA-000731500 |
| E    TGA-000149500 | F    TGA-000080800 |

| G    TGA-002154000 | H    TGA-001611300 |
| --- | --- |
| I    TGA-000024800 | J    TGA-000367600 |

| K    TGA-000478600 | L    TGA-001175900 |
| --- | --- |
| M    TGA-001325800 | N    TGA-002155200 |
| O    TGA-000818700 | P    TGA-000730800 |
| Q    TGA-000739900 | R    TGA-001849400 |
| S    TGA-001506800 |  |
