# Supplementary material for: Multilocus Analysis Resolves the European Finch Epidemic Strain of Trichomonas gallinae and Suggests Introgression from Divergent Trichomonads
Source: Genome Biol Evol. 2019 Jul 30;11(8):2391–402. doi: 10.1093/gbe/evz164 (PMC6735722; doi:10.1093/gbe/evz164)
Supplement: evz164_Supplementary_Data [file evz164_supplementary_data.zip › Supplemental figure_legends.docx]

**Figure S1** **k-mer plots for *T. gallinae* showing high quality assembly statistics. A.** A k-mer histogram showing the frequency distribution of 27-mers from the reads used to estimate the genome size. **B.** The peak of the k-mer histogram (black) compared to a theoretical distribution (Poisson in green). **C.** A k-mer spectra plot comparing the reads to the assembly. The initial peak (black) shows unique or near-unique k-mers from the reads that do not form part of the assembly and are most likely sequencing errors. The second peak (red) shows the majority of the assembled genome. There is very little black in this peak showing that there has been no exclusion of ‘real’ data from the assembly. There is also very little repetition within the assembly suggesting no large-scale repeats that may have impacted the assembly.

**Figure S2. Single locus phylogeny trees for individual genes through A to S.** The Trees were constructed using ML algorithm of MEGA software to each of the MLST loci indicated in Table 4. All markers are able to resolve ribotype A and ribotype C strains as distinct groups, resolution varied within these groups. Trees shown in A, D, and I were observed to be incongruent with respect to the M1 isolate irrespective of outgroup use.

**Figure S3 Constrained and unconstrained ML trees used to test topological significance.** Trees were build using GTR model and Gamma correction in iqtree and rooted with *T. vaginalis*. **A.** Unconstrained tree built using all loci except loci A, D, and I. **B.** Constrained tree built using all loci except loci A, D, and I. Topological constraint forced M1 to be monophyletic with the C clade. **C.** Constrained tree built using all loci except loci A, D, and I. Topological constraint forced M1 to be paraphyletic to the A-C clade. **D.** Unconstrained tree build from alignment of loci A. **E.** Constrained tree build from alignment of loci A. Topological constraint forced M1 to be monophyletic with the C clade. **F.** Constrained tree build from alignment of loci A. Topological constraint forced M1 to be monophyletic with the A clade. **G.** Unconstrained tree built from alignment of loci D. **H.** Constrained tree build from alignment of loci D. Topological constraint forced M1 to be monophyletic with the C clade. **I.** Constrained tree build from alignment of loci D. Topological constraint forced M1 to be paraphyletic with the A-C clade. **J.** Unconstrained tree build from alignment of loci I. **K.** Constrained tree build from alignment of loci I. Topological constraint forced M1 to be monophyletic with the A clade. **L.** Constrained tree build from alignment of loci I. Topological constraint forced M1 to be paraphyletic with the A-C clade
