# Supplementary material for: Multilocus Analysis Resolves the European Finch Epidemic Strain of Trichomonas gallinae and Suggests Introgression from Divergent Trichomonads
Source: Genome Biol Evol. 2019 Jul 30;11(8):2391–402. doi: 10.1093/gbe/evz164 (PMC6735722; doi:10.1093/gbe/evz164)
Supplement: evz164_Supplementary_Data [file evz164_supplementary_data.zip › Table S1_26_11.docx]

**Table S1.** **Gene loci of MLST scheme, Sequences of primers used in the PCR, annealing temperature, length of the obtained amplified fragment**. Nineteen candidate loci screened for MLST scheme for *Trichomonas gallinae.*

| *T. gallinae*  Gene ID | Gene Name | Primer Orientation | Sequences (5' - 3') | Annealing temperature | Sequence  length(bp) | Fig. S1 label |
| --- | --- | --- | --- | --- | --- | --- |
| TGA-000149300 | Arp2/3, putative | Forward  Reverse | GATTATTCTTGAAGCCCACTCC  CTTGCCGGTAACAGTTCTAGC | 55°C | 808 | A |
| TGA-001385000 | Clan MA, family M8, leishmanolysin-like metallopeptidase (GP63a) | Forward  Reverse | ACGGACGAGTGAACAAGCAT  GCCTCGAGATTAGCGTCGAA | 55°C | 682 | B |
| TGA-00112400 | Mismatch repair MutL homolog (MIh1A) | Forward  Reverse | GCTCTGTGGCTCACTGGATT  GTTATGTTGACGATGCCGCC | 57°C | 708 | C |
| TGA-000731500 | Mismatch repair MutL homolog (MIh1A) | Forward  Reverse | TTTGGTGCGCGCATTTCTAC  TTCGCCACGAAAGCCAAATG | 55°C | 966 | D |
| TGA-000149500 | Coronin (CRN) | Forward  Reverse | TCGGGACTTGTAGACCGGAT  AGAAATGGTGCCTGGATGGG | 57°C | 904 | E |
| TGA-000080800 | Antigenic protein P1, putative (VSA) | Forward  Reverse | TTGCCTGCCCCGACTAATTT  CACCAAACGTTGGCACTCAG | 55°C | 741 | F |
| TGA-002154000 | Clan MA, family M8, leishmanolysin-like metallopeptidase (GP63b) | Forward  Reverse | TGCGTGGTGATGCCAATACT  ACGGGGCATATCTGCAACAA | 55°C | 771 | G |
| TGA-001611300 | Vesicular mannose-binding lectin, putative (LLF4) | Forward  Reverse | TGCACTCAGCCGTTTCTCAT  GCAAACAAGGCCAAACGCTA | 55°C | 725 | H |
| TGA-000024800 | Vesicular mannose-binding lectin, putative, PS(LLF1) | Forward  Reverse | TGCACTCAGCCGTTTCTCAT  GCAAACAAGGCCAAACGCTA | 55°C | 736 | I |
| TGA-000367600 | Clan CA, family C1, cathepsin L-like cysteine peptidase | Forward  Reverse | AATGCCCAGCATGATCCACA  ACTCCCAGACACTTTTCGGC | 55°C | 817 | J |
| TGA-000478600 | Clan CA, family C1, cathepsin L-like cysteine peptidase | Forward  Reverse | TGTAAAGCGAGCCCGAAAGT  GGCTGGAAGATCGTAGACGG | 55°C | 721 | K |
| TGA-001175900 | HIV-1 rev binding protein, putative | Forward  Reverse | GGATGCTAATGCACGCACAG  CATGCATGTTTCCGGCGTAG | 57°C | 759 | L |
| TGA-001325800 | Vesicular mannose-binding lectin, putative | Forward  Reverse | TCTGGTCAGCTGGCATCTTG  TGAAGGCGAACGTGCTAACT | 55°C | 910 | M |
| TGA-002155200 | Multidrug resistance pump, putative | Forward  Reverse | GAGAAAATGCGCGCTACAGG  CGACAACAGCGTTAGCATCG | 57°C | 743 | N |
| TGA-000818700 | Aspartic peptidase | Forward  Reverse | TCCTGCTTACCACCGAAACC  TTTCGCCATTGGCAGAGAGT | 55°C | 900 | O |
| TGA-000730800 | Tropomyosin isoforms1/2, putative | Forward  Reverse | GCTGCCAGATGCTGCTAAAC  ATGCAGCCATTCTATCGGCA | 55°C | 655 | P |
| TGA-000739900 | Clan CE, family C48, cysteine peptidase | Forward  Reverse | GCTGCCAGATGCTGCTAAAC  ATGCAGCCATTCTATCGGCA | 55°C | 657 | Q |
| TGA-001849400 | Clan CE, family C48, cysteine peptidase | Forward  Reverse | CATTGCAGCTTTCGTCGTGT  CTCCGCTTAGATGAGCCAGG | 55°C | 867 | R |
| TGA-001506800 | Conserved hypothetical protein (with PF03388 Domain) | Forward  Reverse | TCAATGCCTCTGTTGCCGAT  CTCGTTGCATACGCTGTTCG | 55°C | 700 | S |
