# Supplementary material for: Multilocus Analysis Resolves the European Finch Epidemic Strain of Trichomonas gallinae and Suggests Introgression from Divergent Trichomonads
Source: Genome Biol Evol. 2019 Jul 30;11(8):2391–402. doi: 10.1093/gbe/evz164 (PMC6735722; doi:10.1093/gbe/evz164)
Supplement: evz164_Supplementary_Data [file evz164_supplementary_data.zip › TablesS2andS3.docx]

**Table S2 Results of a Pairwise Homoplasy Index (PHI) testing for evidence of recombination at each MLST locus.**

| T. gallinae gene ID | sig for recombination | p-value |
| --- | --- | --- |
| TGA-000024800.phy | yes | 4.68E-13 |
| TGA-001611300.phy | yes | 3.76E-06 |
| TGA-000367600.phy | yes | 3.84E-06 |
| TGA-00112400.phy | yes | 0.01025 |
| TGA-000080800.phy | yes | 0.01175 |
| TGA-002154000.phy | yes | 0.0458 |
| TGA-000739900.phy | no | 0.1131 |
| TGA-001506800.phy | no | 0.1176 |
| TGA-001849400.phy | no | 0.1197 |
| TGA-000149500.phy | no | 0.1959 |
| TGA-002155200.phy | no | 0.3178 |
| TGA-001385000.phy | no | 0.4069 |
| TGA-001175900.phy | no | 0.4384 |
| TGA-000818700.phy | no | 0.6342 |
| TGA-000730800.phy | no | 0.6677 |
| TGA-000478600.phy | no | 0.6764 |
| TGA-000149300.phy | no | 0.8651 |
| TGA-001325800.phy | no | 0.8679 |
| TGA-000731500.phy | no | 0.8805 |

**Table S3 Results of the topological tests showing all constrained topologies were significantly excluded (p = 0.05).**

| loci | constrained | logL | deltaL | bp-RELL |  | p-KH |  | p-SH |  | c-ELW |  | p-AU |  | Figure reference |
| --- | --- | --- | --- | --- | --- | --- | --- | --- | --- | --- | --- | --- | --- | --- |
| 16 | uncon | -38731.99415 | 0 | 1 | + | 0.999 | + | 1.00E+00 | + | 1 | + | 1 | + | A |
| 16 | con 1 | -40408.17537 | 1676.2 | 0 | - | 0 | - | 0 | - | 0 | - | 1.31E-46 | - | B |
| 16 | con 2 | -38768.98023 | 36.986 | 0.0001 | - | 0.0009 | - | 0.289 | + | 9.97E-05 | - | 0.00015 | - | C |
| A | uncon | -2776.675641 | 0 | 0.867 | + | 0.867 | + | 1 | + | 0.83 | + | 0.863 | + | D |
| A | con 1 | -2780.048974 | 3.3733 | 0.133 | + | 0.133 | + | 0.215 | + | 0.166 | + | 0.151 | + | E |
| A | con 2 | -2785.839737 | 9.1641 | 0.0007 | - | 0.0126 | - | 0.0138 | - | 0.00458 | - | 0.00072 | - | F |
| D | uncon | -2952.720935 | 0 | 0.999 | + | 0.999 | + | 1 | + | 0.998 | + | 0.999 | + | G |
| D | con 1 | -2974.320658 | 21.6 | 0.0006 | - | 0.0013 | - | 0.0013 | - | 0.00114 | - | 0.00051 | - | H |
| D | con 2 | -2974.320584 | 21.6 | 0.0008 | - | 0.0013 | - | 0.0013 | - | 0.00114 | - | 0.00057 | - | I |
| I | uncon | -3127.583955 | 0 | 0.987 | + | 0.981 | + | 1 | + | 0.984 | + | 0.993 | + | J |
| I | con 1 | -3150.861435 | 23.277 | 0.0064 | - | 0.0195 | - | 0.0195 | - | 0.00781 | - | 0.00739 | - | K |
| I | con 2 | -3150.860265 | 23.276 | 0.0063 | - | 0.0195 | - | 0.0195 | - | 0.00782 | - | 0.00728 | - | L |
|  |  |  |  |  |  |  |  |  |  |  |  |  |  |  |
| deltaL : logL difference from the maximal logl in the set. | | | | | | |  |  |  |  |  |  |  |  |
| bp-RELL : bootstrap proportion using RELL method (Kishino et al. 1990). | | | | | | | | |  |  |  |  |  |  |
| p-KH : p-value of one sided Kishino-Hasegawa test (1989). | | | | | | |  |  |  |  |  |  |  |  |
| p-SH : p-value of Shimodaira-Hasegawa test (2000). | | | | | |  |  |  |  |  |  |  |  |  |
| c-ELW : Expected Likelihood Weight (Strimmer & Rambaut 2002). | | | | | | | |  |  |  |  |  |  |  |
| p-AU : p-value of approximately unbiased (AU) test (Shimodaira, 2002). | | | | | | | | |  |  |  |  |  |  |
| Plus signs denote the 95% confidence sets. | | | |  |  |  |  |  |  |  |  |  |  |  |
| Minus signs denote significant exclusion. | | | |  |  |  |  |  |  |  |  |  |  |  |
| All tests performed 10000 resamplings using the RELL method. | | | | | | |  |  |  |  |  |  |  |  |
