# Supplementary figures and images for: Multilocus Analysis Resolves the European Finch Epidemic Strain of Trichomonas gallinae and Suggests Introgression from Divergent Trichomonads
Source: Genome Biol Evol. 2019 Jul 30;11(8):2391–402. doi: 10.1093/gbe/evz164 (PMC6735722; doi:10.1093/gbe/evz164)

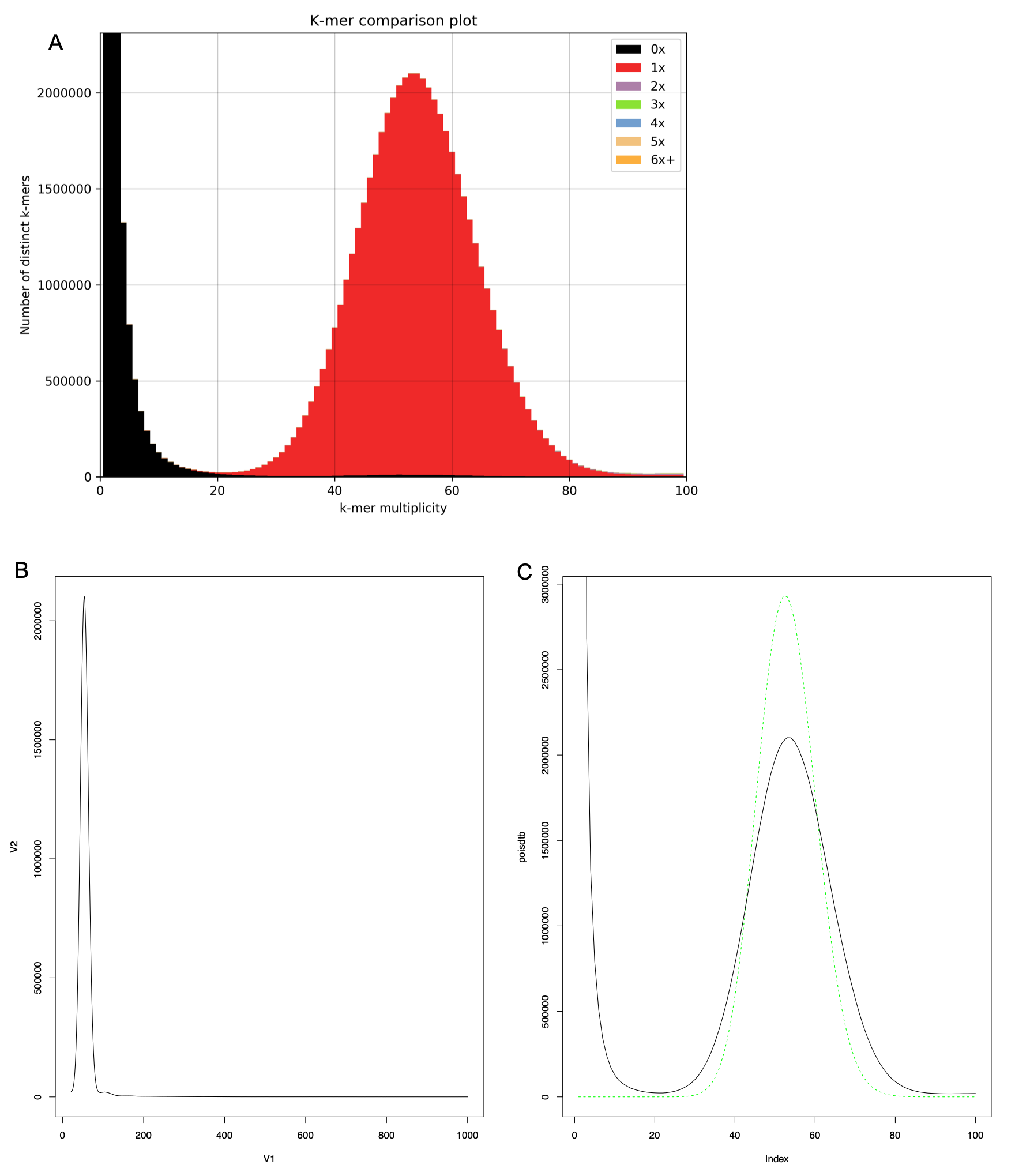

Supplement: evz164_Supplementary_Data [file evz164_supplementary_data.zip › Figure_S1.png]

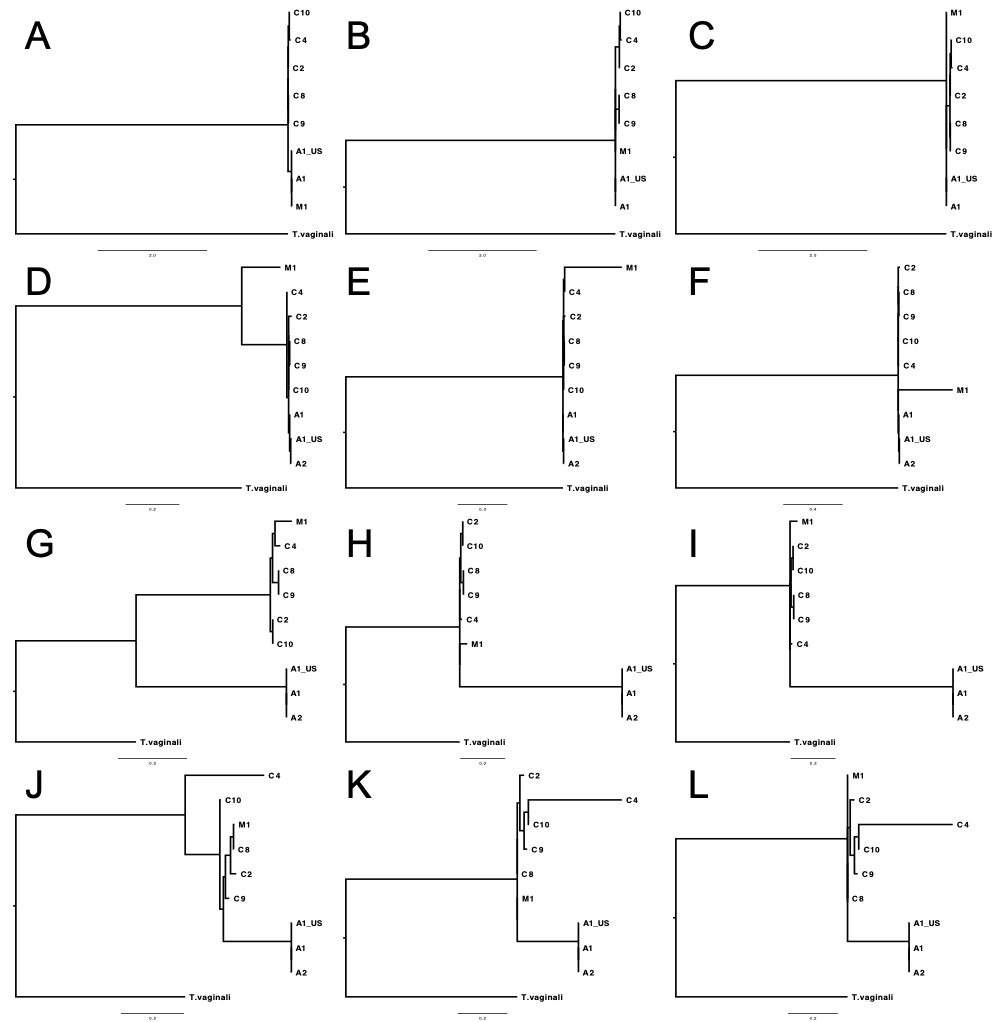

Supplement: evz164_Supplementary_Data [file evz164_supplementary_data.zip › Figure_S3.png]
